# Relationship Between Finger Photoplethysmographic Pulses and Skin Blood Perfusion

**DOI:** 10.7759/cureus.71035

**Published:** 2024-10-07

**Authors:** Harvey N Mayrovitz

**Affiliations:** 1 Medical Education, Nova Southeastern University Dr. Kiran C. Patel College of Allopathic Medicine, Davie, USA

**Keywords:** laser doppler, ldf, oxygen saturation, perfusion index, photoplethysmography, ppg, pulsatility index, skin blood perfusion, spo2

## Abstract

Introduction

Photoplethysmography (PPG) measures are important in monitoring peripheral oxygen saturation (SpO_2_). Another parameter is a derived quantity referred to as the peripheral perfusion index (PPI). It is calculated as the ratio of the peak-to-peak pulse amplitude of a PPG signal (PA_PPG_) to the non-pulsating part of the total PPG signal. The PPI has been used as a marker of blood perfusion states in a variety of clinical settings but has not been systematically and directly compared to measures of local blood perfusion. This study’s purpose was to investigate this issue to provide initial data on the relationship between finger skin blood perfusion, measured by laser Doppler blood perfusion flux (LDF) and PA_PPG_.

Methods

Ten subjects (five male), recruited from medical students with an average age of 26 years, participated. While supine for 30 minutes, skin blood perfusion was recorded using laser Doppler flux (LDF) on the ring finger pulp of the non-dominant hand, and the photoplethysmography pulse (PPG) was recorded from the index finger of the same hand. The recorded data was searched sequentially manually to locate the first 30-pulse sequence in which the PPG amplitude of at least six PPG pulses was less than or equal to 60% of the maximum pulse amplitude in the sequence. The primary PPG parameter of interest was PA_PPG_. For the LDF signal, the pulse amplitude is designated as PA_LDF_, the total LDF for each pulse is designated as LDF_TOT, _and the LDF pulsatile component is designated as PF. To investigate the relationship between LDF parameters and PA_PPG_ a linear regression analysis of each 30-pulse sequence was done with PA_PPG_ as the independent variable and each of the three LDF parameters individually (PA_LDF_, LDF_TOT_, and PF) as dependent variables.

Results

There was a statistically significant direct relationship between PA_PPG_ and all three measures of blood perfusion (p<0.05). Correlation coefficients (R) varied among subjects but within-subject variations versus PA_PPG_ were similar, having mean values that ranged from 0.665 to 0.694. The results also provided evidence in support of a direct relationship between the LDF pulsatility index, defined as the ratio of PF to its mean value., and PA_PPG_ (R=0.779).

Conclusions

When finger PPG pulse amplitudes are measured in individual subjects there is a moderate-to-strong correlation between the PPG pulse amplitude changes and skin blood perfusion changes. This fact impacts the confidence in using the widely available PPG parameter, peripheral perfusion index, as an indicator of changes in tissue perfusion. However, differences in the PPG pulse amplitude among subjects were less reliable indicators of differences in blood perfusion among subjects. The findings also indicate that a related parameter, the LDF pulsatility index, is also highly correlated with the PPG pulse amplitude and may serve as a useful parameter for future clinical investigations.

## Introduction

Photoplethysmographic (PPG) assessments have an important place in monitoring peripheral oxygen saturation (SpO_2_) in multiple settings and conditions [1,2]. Frequently such assessments are done using a sensor placed on a finger to measure SpO_2_ and other newly implemented parameters such as blood pressure [3,4] and pulse wave velocity [5,6]. An additional parameter now available in most devices is a derived quantity referred to as the peripheral perfusion index (PPI). It is calculated as the ratio of the peak-to-peak pulse amplitude of a PPG signal (PA_PPG_) to the non-pulsating part of the total PPG signal [7]. The PPI is not to be confused with the pulsatility index (PLI) which is a parameter expressed as the ratio of pulsatile blood velocity to the corresponding mean or a reference value and used as a parameter to characterize the extent of peripheral arterial disease [8,9] and also used for a range of other conditions related to heart, pulmonary and cerebral features [10-12].

Contrastingly, initial work on the PPI was targeted to investigate its use it as an indicator of tissue perfusion using its value compared to that of the core-to-toe skin temperature difference (DT) in a group of 37 patients [13]. A DT ≥ 7^o^C was arbitrarily defined as an abnormal overall perfusion. According to this criterion, measurements in the same patients at times when they had normal vs. abnormal perfusion were reported. Based on the generally lower PPI value these researchers found during the abnormal perfusion times, it was suggested that PPI might be a good indicator of tissue perfusion. Because low perfusion states may impact the accuracy and precision of SpO_2_ measurements [14,15], a more recent study used a PPI value of ≤1% as a criterion to indicate low perfusion in persons with Fitzpatrick classes from I through VI [16]. Based on measurements using two different finger PPG devices, these researchers concluded that both PPI and darker skin were associated with clinically significant high-reading errors. The possibility of using PPI as a detector of, or discriminator for, low perfusion states has also been evaluated for a specific device [17]. In this study a PPI value of 1% was used as an arbitrary cutoff value and SpO_2_ values were reported accurate for both normal and low perfusion values independent of skin tone. 

Although the PPI value may be construed to be at least in part an indicator of tissue blood perfusion an important unanswered question is the extent to which PA_PPG_, the main variable in the PPI value [7], is related to actual pulsatile tissue blood perfusion. The purpose of the present pilot study was to investigate this issue to provide initial data on the extent of the relationship between finger skin blood perfusion, measured by laser Doppler blood perfusion flux (LDF) and PA_PPG_.

## Materials and methods

Subjects

Ten volunteer subjects (five male), recruited from 1st and 2nd-year medical students, participated in this research after signing a University Institutional Review Board (IRB) approved consent form (#2019-598-NSU). Entry requirements were (1) the ability and agreement to lie supine without significant movements for up to 45 minutes, (2) no history of cardiovascular or neurological conditions, (3) nonsmoker, and (4) willingness to forgo caffeinated beverages on the day of the experiment. Persons with diabetes mellitus were excluded from participation.

Measurements

**Figure 1:**
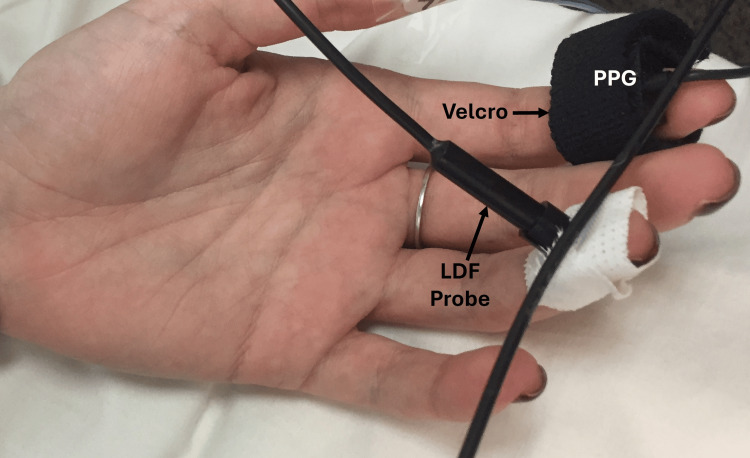
Measurement setup Laser Doppler flux (LDF) is measured on the pulp of the ring finger using a multifiber LDF probe. The photoplethysmographic (PPG) signal is obtained from the index finger with the PPG sensor secured to the finger firmly but gently with Velcro.

Skin blood perfusion was measured using laser Doppler flux (LDF) measurements with a multifiber laser integrating probe (Moor DP7A, Moor Instruments, Wilmington DE, USA) attached to the ring finger pulp of the non-dominant hand as shown in Figure 1. The probe was connected to a laser Doppler measuring system (Laserflo Blood Perfusion Monitor, Moor Instruments, Model BPM2). The recorded blood perfusion or flux is the product of the red blood cell (RBC) concentration and RBC velocity. The measurement uses a low-intensity laser light signal that is transmitted into the skin to a depth of about 1-2 mm [18]. The Doppler-shifted return signal contains information about the speed and number density of moving RBCs, which is processed to yield a parameter, RBC perfusion or RBC flux, that is proportional to blood flow. The system is calibrated using a motility standard provided by the company. LDF is expressed as relative units since it cannot be directly expressed in blood flow units and is widely used to measure skin blood perfusion [19-22]. The PPG pulse was recorded from the index finger of the same hand using a matched infrared emitter and photodiode sensor (TSD200, Biopac Systems, Goleta CA USA) that was gently but securely secured to the finger with Velcro as also illustrated in Figure 1. The system works with an infrared excitation of 860 ± 60 nm with a detected wavelength of 800 nm. Blood pulses in the finger tissue modulate the infrared causing changes in the resistance of the sensor producing a time-varying output voltage reflecting these pulsations. The PPG sensor output was coupled to a PPG amplifier (PPG100C, Biopac Systems, Goleta CA USA) that was set to a gain of 50, a low pass filter of 10 Hz, and a high pass filter of 0.05 Hz. The outputs of the PPG amplifier and the laser Doppler system were coupled to an analog/digital conversion device (DataQ Instruments, Akron OH, USA, model DI-720), and each channel was sampled at 1000 samples per second using Windaq recording and playback software (DataQ Instruments, Akron OH, USA) and the two output channels displayed and recorded on a laptop computer. The channel gains were both set to four for all experiments. 

Measurement procedures

With the subject supine on a padded examination table, the laser Doppler probe and the PPG sensor were placed on the target fingers as illustrated in Figure 1. Afterward, the room lights were dimmed, and the two channels were observed for amplitude suitability at the set gain. The gain remained at a value of four for all subjects. After the subject had been lying supine for 10 minutes, the recording was started, and data was acquired for 30 continuous minutes. At the end of the recording interval, the subject’s blood pressure was taken and recorded while they were still supine.

Data extraction

**Figure 2:**
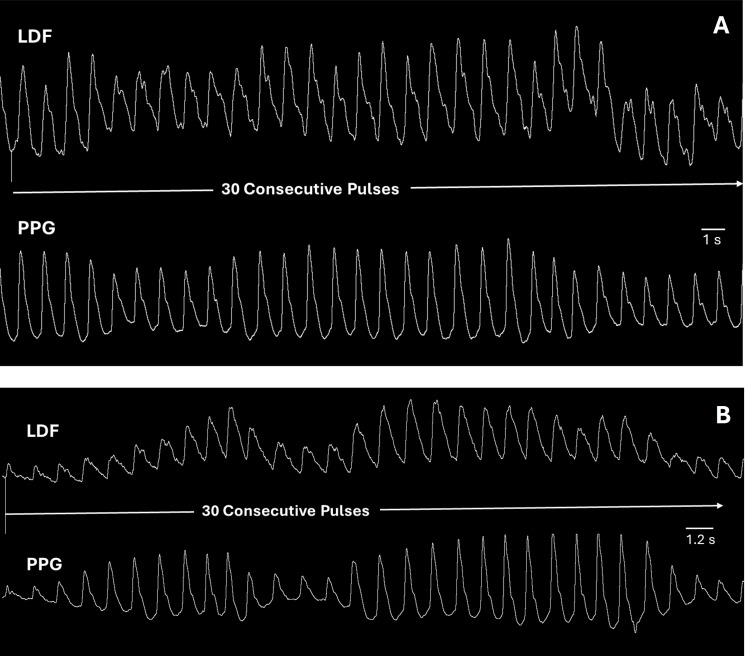
Example measurement sequences LDF is the laser Doppler flux measured on the pulp of the ring finger. PPG is the photoplethysmography signal from the index finger.  Both are shown as a function of time. In A, the 30-pulse sequence that was analyzed was captured 1496 seconds into the experiment. In B. the 30-pulse sequence that was analyzed was captured 4.5 seconds into the experiment. In both cases there is a spontaneous waxing and waning of the PPG signal with associated changes in the pattern of the LDF signal.

The recorded data was searched sequentially manually to locate the first 30-pulse sequence in which the PPG amplitude of at least six PPG pulses was less than or equal to 60% of the maximum pulse amplitude in the sequence. Two such sequences are illustrated in Figure 2. In one of the data sets such a sequence was not observed until 1496 seconds (~ 25 minutes) from the start of the recording. In the other example data set a sequence satisfying the search criterion was located after only 4.5 seconds from the start of the recording. The reason for the 30-pulse criterion was to avoid potential temporal confounding factors of longer intervals while still including enough pulses to provide a reasonable regression estimate. The criterion of at least six pulses with pulse amplitudes ≤ the maximum pulse amplitude in that interval was to have a sufficient range of pulse amplitudes for a meaningful regression analysis.

Data parameters

**Figure 3:**
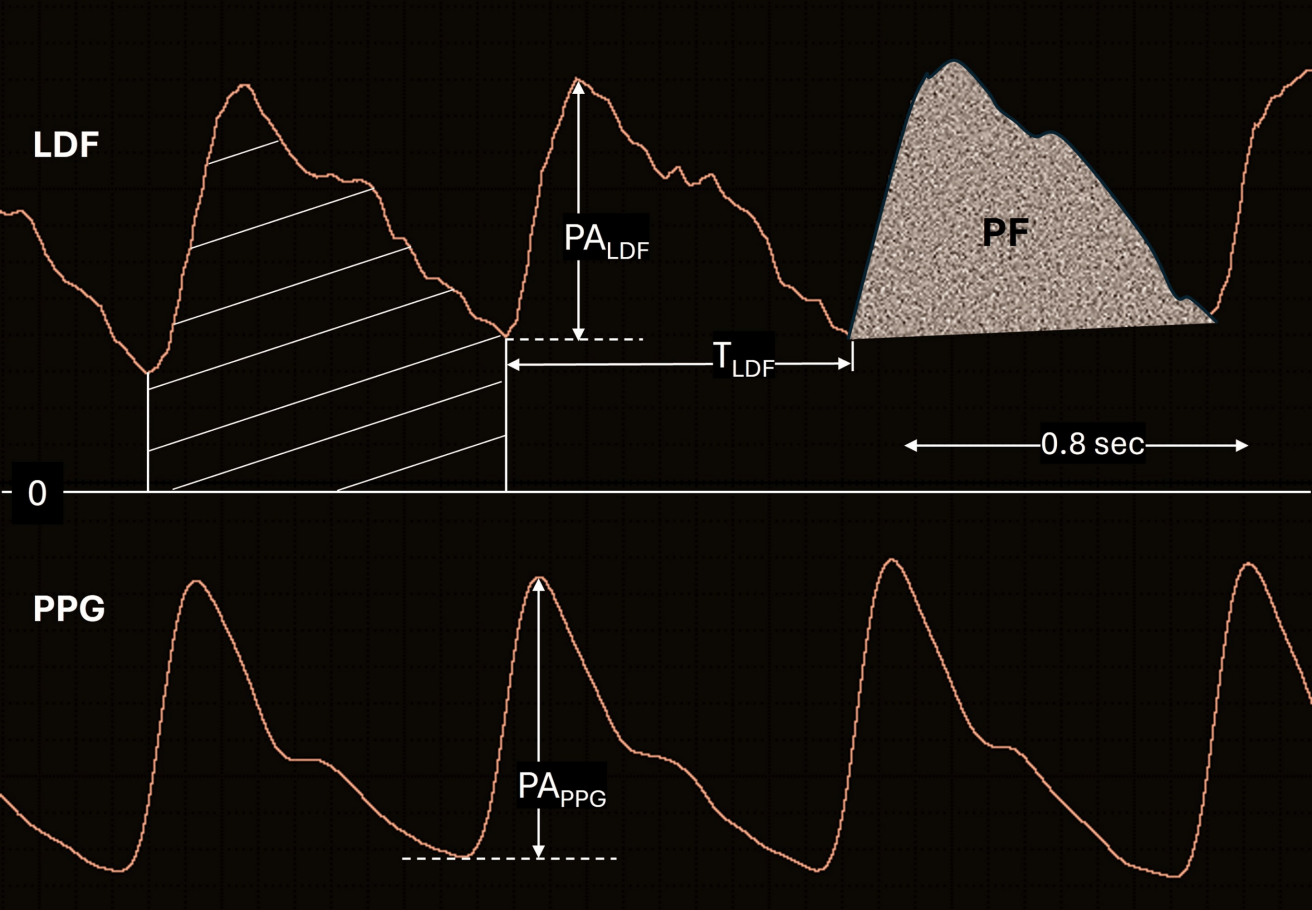
Parameter definitions LDF is the laser Doppler flux measured on the pulp of the ring finger. PPG is the photoplethysmography signal recorded from the index finger of the same hand.  Both are shown as a function of time. PALDF is the peak-to-peak amplitude of the LDF and PAPPG is the peak-to-peak amplitude of the PPG pulse. TLDF is the duration of the LDF pulse. The area with the diagonal lines in the LDF signal is the total LDF per pulse (LDFTOT). The area enclosed by the pulsatile part and labeled PF is the pulsatile flux component.

The primary PPG parameter is the PPG pulse amplitude which is the peak-to-peak value illustrated in Figure 3 and as previously described is denoted as PA_PPG_. For the LDF signal, also illustrated in Figure 3, the pulse amplitude is designated as PA_LDF_. The total LDF associated with each pulse is represented by the total area under the curve and is designated as LDF_TOT_. The pulsatile component of the LDF is designated as PF.

Analysis

To investigate the relationship between LDF and PA_PPG_ a linear regression analysis of each 30-pulse sequence was done with PAPPG as the independent variable and each of the three LDF parameters individually (PA_LDF_, LDF_TOT_, and PF) as dependent variables. For each regression parameter, the best and the least good correlation was determined based on the least and greatest correlation coefficient (R), and the average correlation coefficient among all 10 subjects was determined. The overall relationship of perfusion parameters to PA_PPG_ was determined via regression analysis based on the combined data of the 300 measured pulses. The possible relationship between the LDF pulsatility index (PLI), defined as the ratio of PA_LDF_ to its mean value (MEAN_LDF_), was determined by regression analysis of each subject’s average PLI with each subject’s average PA_PPG_. P-values < 0.05 were taken as statistically significant.

## Results

Subject demographics

Table 1 lists the key parameters for each of the 10 subjects. Based on body mass index (BMI) values, four of the male subjects would be classified as slightly overweight (BMI > 25.0 Kg/m^2^) with all others classified as normal weight. The blood pressure of four of the subjects would be classified as elevated based on the current American Heart Association standards due to their systolic pressures being not less than 120 mmHg. However, in the present study subject blood pressures were measured during supine lying, not in a seated position as is the standard. Diastolic pressures of all subjects were within the normal range. For the entire group, average values ± SD for age, BMI, systolic BP, diastolic BP, and heart rate were respectively 26.0 ± 1.3 years, 23.2 ± 2.7 Kg/m_2_, 117.6 ± 7.7 mmHg, 69.6 ± 6.1 mmHg, and 65.6 ± 10.8 bpm.

**Table 1:** . Participant demographics Based on body mass index (BMI) values, four of the male subjects (5, 6, 8 and 9) would be classified as slightly overweight (BMI > 25.0). Blood pressure (BP) and heart rate were measured during supine lying. According to current American Heart Association standards, four subjects would be classified as having elevated BP (subjects 2, 6, 9, and 10).

Subject	Sex	Age (years)	Height (m)	Weight (Kg)	BMI (Kg/m^2^)	Systolic BP (mmHg)	Diastolic BP (mmHg)	Heart Rate (bpm)
1	M	28	1.73	69.2	23.6	116	65	69
2	F	26	1.65	61.6	23	128	77	56
3	M	26	1.63	64.7	24.9	104	60	69
4	F	24	1.65	53.6	20.1	107	68	78
5	M	26	1.73	75.9	25.8	119	63	55
6	M	26	1.70	71.4	25.1	120	69	54
7	F	26	1.73	58.0	19.8	116	72	78
8	F	28	1.78	80.4	25.8	117	76	51
9	M	26	1.80	80.4	25.1	121	68	68
10	F	24	1.60	47.3	18.8	128	78	79

LDF vs. PPG pulse amplitudes

**Figure 4:**
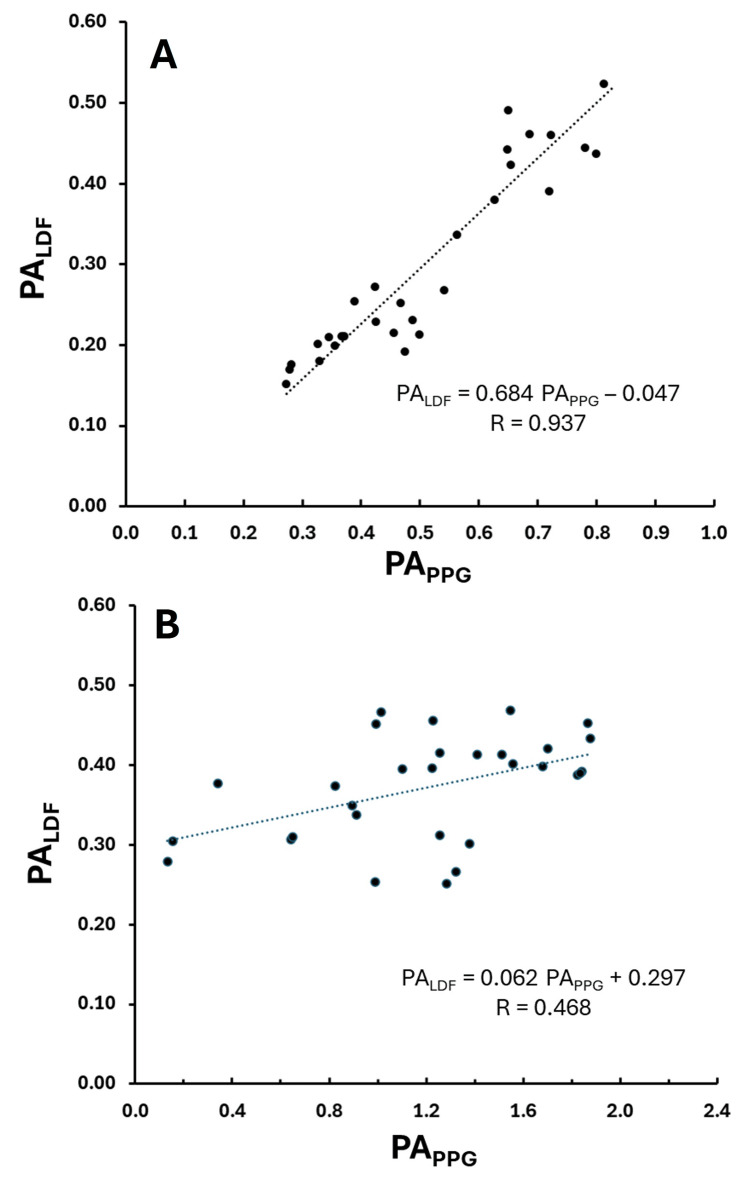
Best and worst correlations for LDF pulse amplitude The pulse amplitude of the laser Doppler flux (PALDF) is shown versus the pulse amplitude of the PPG (PAPPG) along with the linear regression line (dotted) and corresponding regression equation. Part A is the best correlation among the 10 subjects (R = 0.937) and part B shows the least good correlation among the 10 subjects (R = 0.468). Data is for the sequence of 30 pulses, and R is the correlation coefficient.

Figure 4 depicts the relationship between PA_LDF_ and PA_PPG_ for the data representing the best correlation coefficient (R) case (R = 0.937) and the least good correlation case (R = 0.468) among the 10 subjects listed in Table 1. The best correlation was for subject 10 (p < 0.00001) and the least good, but still statistically significant, for subject 9 (p=0.01). The overall average value for R across the 10 subjects (mean ± SD) was 0.694 ± 0.156.

Total LDF pulsatile perfusion vs. PPG pulse amplitude

**Figure 5:**
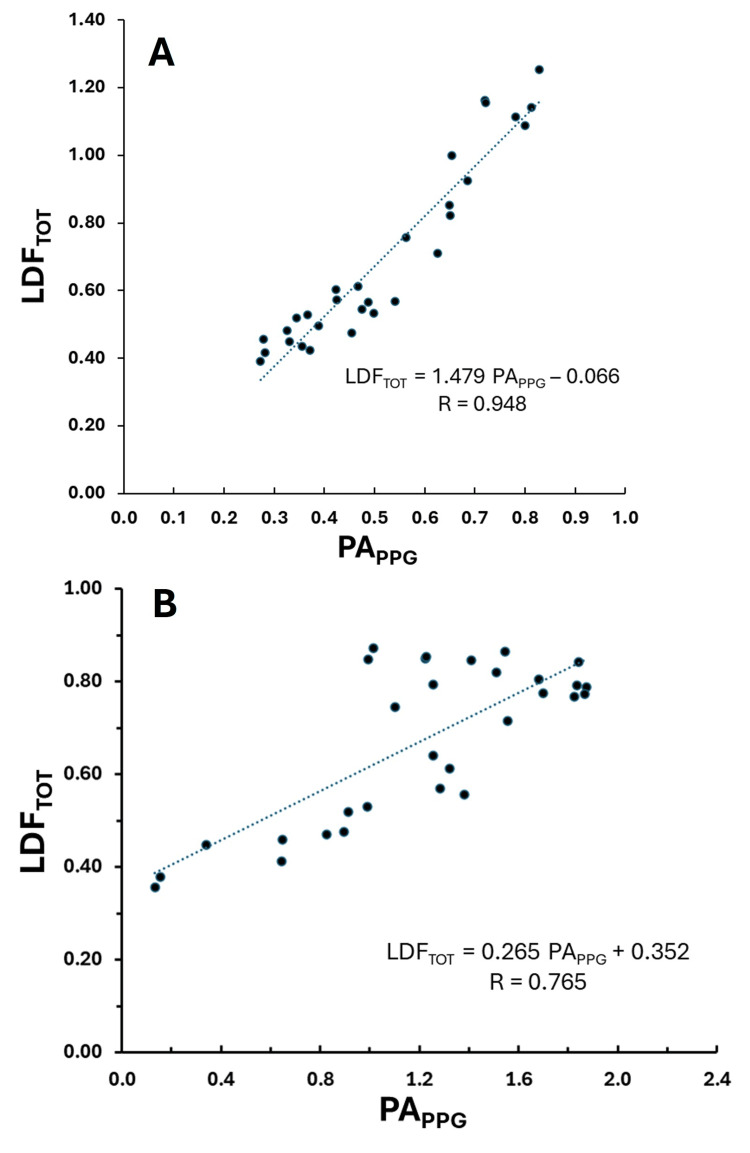
Best and worst correlations for total LDF The total LDF per pulse (LDFTOT) is shown versus the pulse amplitude of the PPG (PAPPG) along with the linear regression line (dotted) and corresponding regression equation for the same two subjects (A and B) as shown in Figure 4. Data is for the sequence of 30 pulses, and R is the correlation coefficient.

Figure 5 depicts the relationship between total LDF (LDF_TOT_) and PA_PPG_ for the data representing the same two subjects shown in Figure 4. The correlation coefficient (R = 0.948) for subject 10 was the best among all 10 subjects (p < 0.00001). The correlation for subject 9 was less good (R = 0.765) but still highly statistically significant (p<0.0001). R-values across the 10 subjects for this relationship ranged from 0.317 for subject 5 (p = 0.039) to 0.948 (p< 0.0001) for subject 10. The overall average R across the 10 subjects (mean ± SD) was 0.695 ± 0.226.

LDF pulsatile perfusion component vs. PPG pulse amplitude

**Figure 6:**
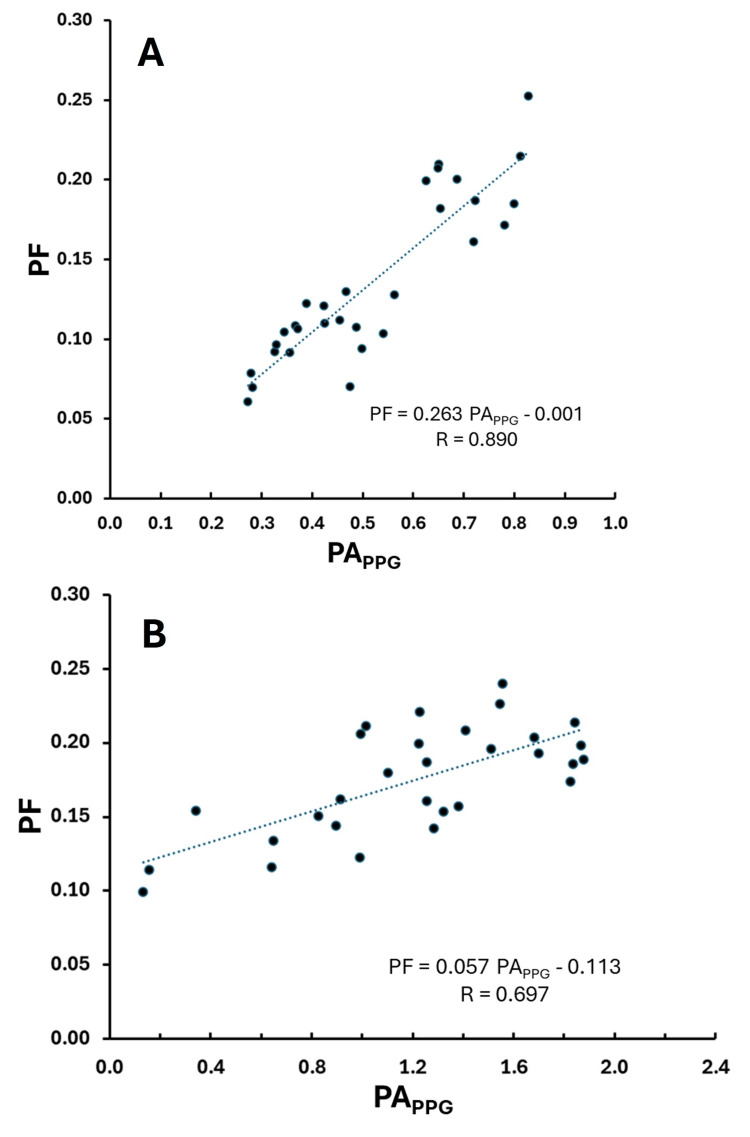
Best and worst correlations for pulsatile component of LDF The pulsatile flow component (PF) is shown versus the pulse amplitude of the PPG (PAPPG) along with the linear regression line (dotted) and corresponding regression equation for the same two subjects (A and B) as shown in Figure 4. Data is for the sequence of 30 pulses, and R is the correlation coefficient. Subjects A and B are the same as shown in Figure 4.

Figure 6 depicts the relationship between the pulsatile component of LDF (PF) and PA_PPG_ for the same two subjects as shown in Figures 4, 5. The correlation coefficient (R = 0.890) for subject 10 was the best among all 10 subjects (p < 0.00001). The correlation for subject 9 was less good (R = 0.697) but still statistically significant (p = 0.0003). R-values across the 10 subjects for this relationship ranged from 0.257 (p =0.112) for subject 5 to 0.889 ( p < 0.0001) for subject 10. The overall average value for R across the 10 subjects (mean ± SD) was 0.665 ± 0.183.

The overall correlation between LDF and PPG pulse amplitudes

**Figure 7:**
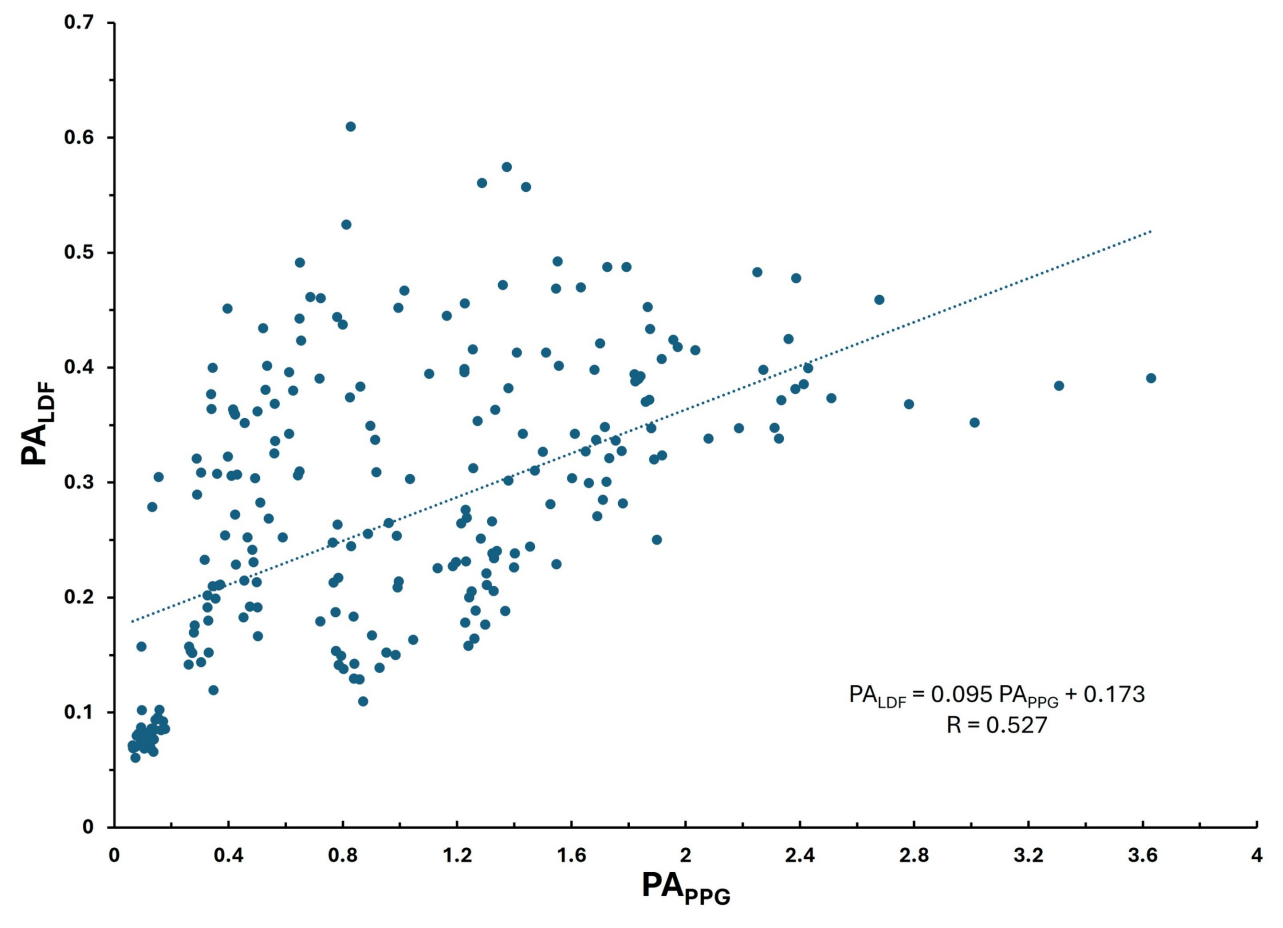
Correlation among all subjects for LDF pulse amplitude The pulse amplitude of the laser Doppler flux (PALDF) is shown versus the pulse amplitude of the PPG (PAPPG) along with the linear regression line (dotted) and corresponding regression equation for all subjects. The p-value for the regression is < 0.0001.

Figure 7 shows the overall composite relationship between PA_LDF_ and PA_PPG_ considering all subject data. Each data point represents a single PA_LDF_ - PA_PPG_ pair (n =300 pulses). A moderate correlation is observed (R = 0.527) that is statistically significant (p< 0.0001). The 95% confidence limits (lower and upper bounds) on the intercept and slope of the regression equation shown in the figure are for the intercept 0.151 to 0.196 and the slope 0.077 to 0.114.

The overall correlation between PF and PPG pulse amplitudes

**Figure 8:**
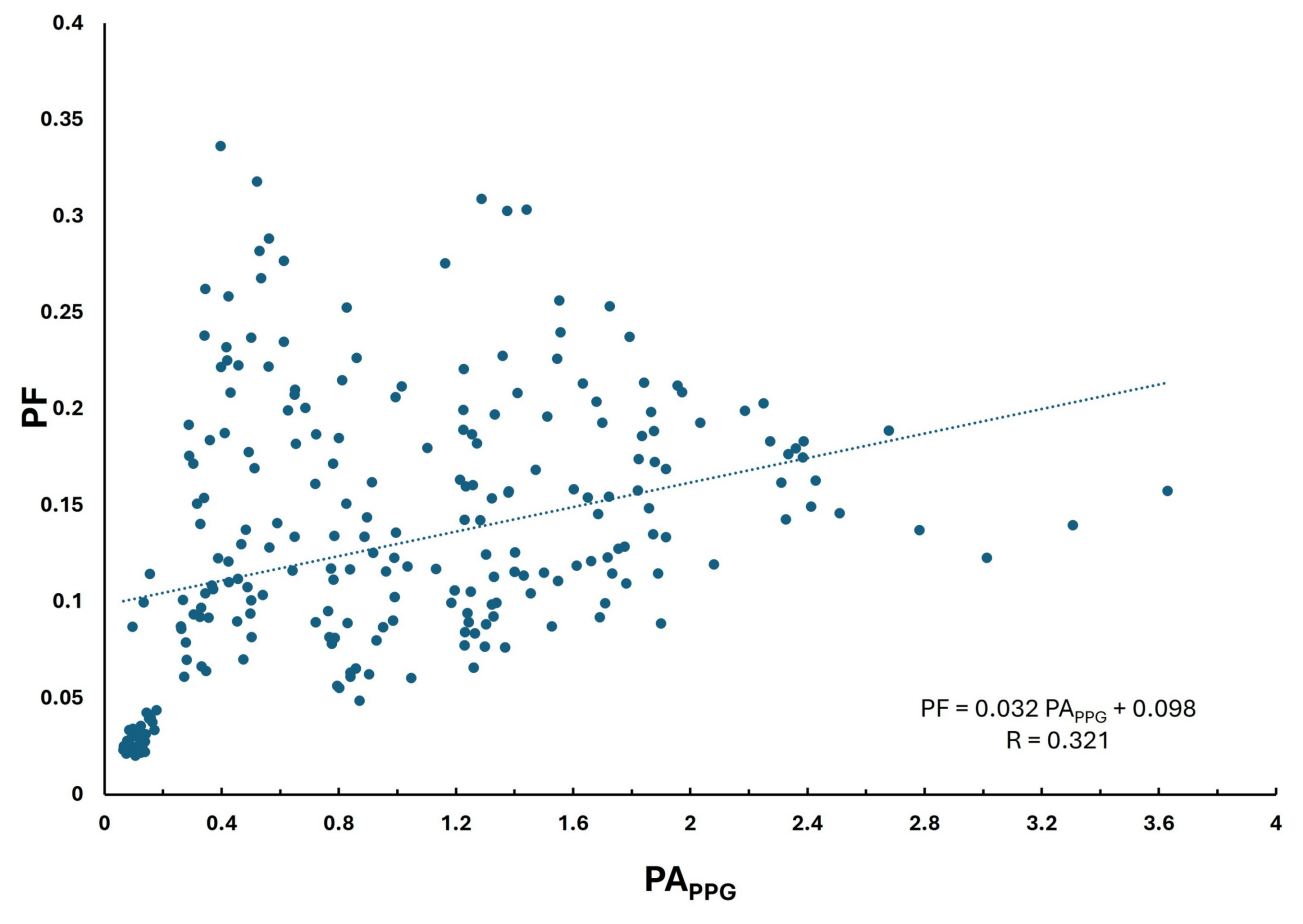
Correlation among all subjects for just the pulsatile LDF component The pulsatile flow component (PF) is shown versus the pulse amplitude of the PPG (PAPPG) along with the linear regression line (dotted) and corresponding regression equation for all subjects. The p-value for the regression is < 0.0001.

Figure 8 shows the overall composite relationship between PF and PA_PPG_ considering all subject data. Each data point represents a single PF - PA_PPG_ pair (n =300 pulses). A low correlation is observed (R = 0.321) but is statistically significant (p< 0.0001). The 95% confidence limits (lower and upper bounds) on the intercept and slope of the regression equation shown in the figure are for the intercept 0.084 to 0.112 and the slope, 0.021 to 0.043.

Overall blood perfusion comparison

**Figure 9:**
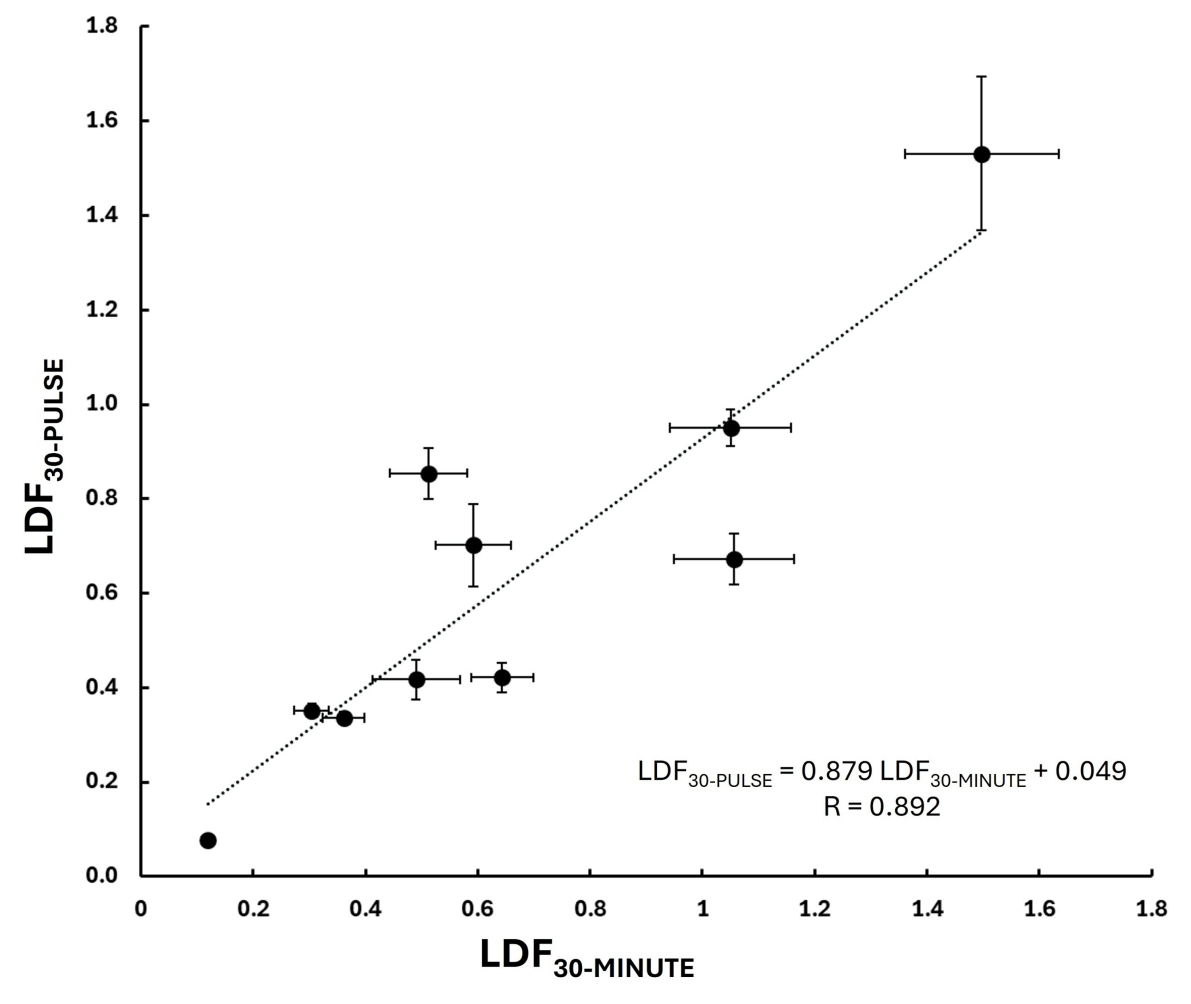
Average blood perfusion comparison Average LDF during the 30-pulse sampling interval (LDF30-PULSE) in relation to the overall 30-minute average LDF perfusion (LDF30-MINUTE) is shown for each of the 10 subjects. The dotted line is the linear regression with the equation shown in the inset. The p-value of the regression is < 0.001. Error bars are the standard error of the mean (SEM).

Figure 9 shows the average LDF during the 30-pulse sampling interval (LDF_30-PULSE_) for each of the 10 subjects compared to the overall 30-minute average LDF perfusion (LDF_30-MINUTE_) for that subject. The high correlation between these two (R = 0.892, p < 0.001) is consistent with the notion that the 30-pulse sample is reflective of the overall perfusion feature. The 95% confidence limits (lower and upper bounds) on the intercept and slope of the regression equation shown in the figure are for the intercept -0.232 to 0.330 and for the slope, 0.515 to 1.242. The 95% confidence limits (lower and upper bounds) on the intercept and slope of the regression equation shown in the figure are for the intercept 0.089 to 0.528 and the slope, 0.059 to 0.453.

LDF pulsatility index

**Figure 10:**
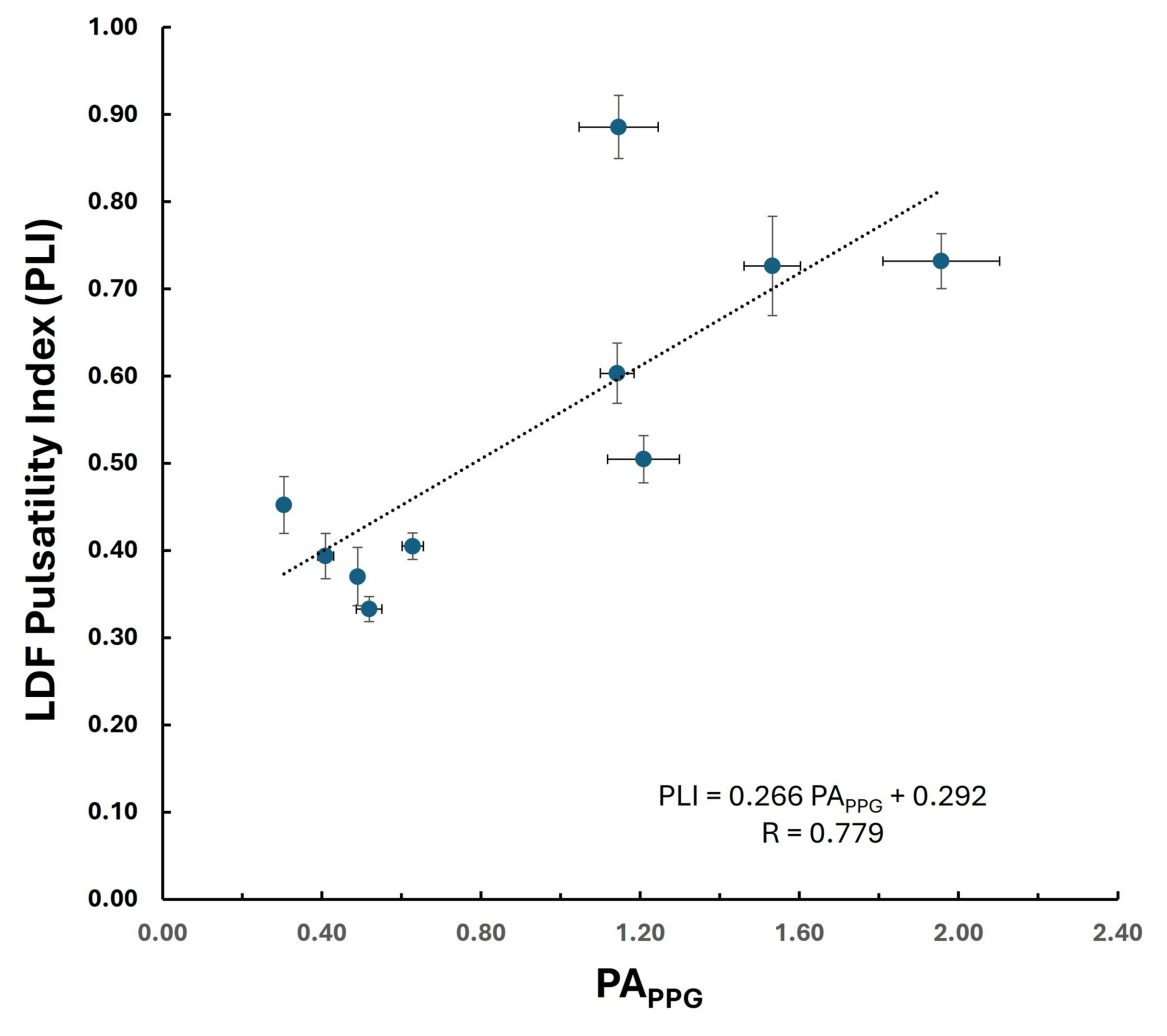
LDF pulsatility index The LDF pulsatility index (PLI = PALDF/MEANLDF) is shown for each of the 10 subjects compared to their PAPPG averaged over their 30-pulse sampling interval. The dotted line is the linear regression with the equation shown in the inset. The p-value of the regression = 0.014. Error bars are the standard error of the mean (SEM).

Figure 10 shows the LDF pulsatility index (PLI = PA_LDF_/MEAN_LDF_) for each of the 10 subjects compared to their peak-to-peak PPG pulse amplitude (PA_PPG_) averaged over each subject’s 30-pulse sampling interval. The data indicates a significant correlation between the pulsatility index and the PPG pulse amplitude (R = 0.779, p = 0.014).

## Discussion

The present study findings appear to be the first to provide some direct evidence of a statistically significant direct relationship between the amplitude of the finger pulse PPG signal and measures of skin blood perfusions determined by laser Doppler measurements. The skin perfusion measures evaluated included the LDF pulse amplitude (PA_LDF_), total LDF perfusion (LDF_TOT_), and the LDF pulsatile component (PF). The correlation coefficients for each parameter varied among subjects ranging from a nonsignificant low of 0.257 in one subject to a high of 0.948 in another. However, the overall correlation coefficients among these three LDF measures across the 10 subjects evaluated were similar and had mean values that ranged from 0.665 to 0.694. Thus, the overall correlations could be considered moderate to strong [23]. In addition, the present results also for the first time provide evidence in support of a direct relationship between the LDF pulsatility index and the amplitude of the PPG finger pulse. This relationship is characterized by a strong correlation (R = 0.779) indicating a positive direct relationship. The strong correlations apply when considering the dependence of the perfusion parameters on the PPG pulse amplitude in individual subjects. However, when considering the correlations among subjects the correlation is less good as exemplified by the regressions shown in Figures 7, 8. One interpretation of these combined findings is that changes in the PPG pulse amplitude reflect the same directional changes in the skin blood perfusion parameters but that differences in the PPG pulse amplitude among subjects are less reliable indicators of differences in blood perfusion among subjects.

In addition to the new insight into the physiological relationships between PPG pulse amplitude signals and the several blood perfusion parameters herein identified; the present findings may have direct clinically related impacts. One relates to the clinical use of the peripheral perfusion index obtained with PPG-determined SpO_2_ in patients. The PPI is often stated as being mainly dependent on stroke volume and vascular tone [24]. The implication would be that lower values of either would be associated with lower PPI values. This concept is supported by several clinical studies, although until now direct systematic comparisons linking PPG amplitude to blood perfusion are lacking. PPI values obtained from a single device and their variation among fingers in 391 healthy subjects indicated a range between 3.54% to 4.66% with the middle right finger yielding the largest mean value [25]. PPI measurements in 20 healthy volunteers who experienced simulated blood volume reduction via lower body negative pressure demonstrated reduced PPI values in response [26]. Subjects with higher baseline PPI values produced the greatest reduction presumably due to peripheral vasoconstriction that was even greater when combined with pain induced by opposite-hand cold water immersion. The PPI has also been used to estimate its association with postoperative acute kidney injury [27]. The strongest association appeared to be with PPI values less than 1%. Changes in PPI relative to changes in blood pressure (BP), stroke volume (SV), and cardiac output (CO) during anesthesia induction suggested that PPI values that decrease vs. those that increase during induction may discriminate between large and smaller decreases in BP, SV, and CO during the induction process [28]. Moreover, the results of PPI monitoring of 291 high-risk patients indicated that a low PPI value was associated with serious adverse events [29]. Contrastingly an increase in PPI during epidural anesthesia was reported to be an earlier indicator of sympathectomy effectiveness than either skin temperature or blood pressure changes [30]. These reported clinical correlations are consistent with the present findings directly linking blood perfusion to the PPG pulse amplitude which is the main factor impacting PPI.

A related parameter studied was the LDF pulsatility index defined as the ratio of the LDF pulse component to the LDF mean value and its relationship to the PPG pulse amplitude. The broader use of the pulsatility index has had several clinical uses. One is the pulmonary artery pulsatility index (PAPi) which is defined as the ratio of the pulmonary artery pulse pressure to right atrial pressure [31], and has been used as a predictor of right ventricular failure [32] and as an indicator of right ventricular function following heart surgery [33]. Another type and use of a pulsatility index is related to cerebral small vessel disease [11,34]. For this application, the pulsatility index is determined by transcranial Doppler ultrasound of the middle cerebral artery, and the pulsatility index is defined as the blood velocity pulse amplitude divided by the mean blood velocity. A large pulse index was reported to be associated with greater small vessel compromise, a greater incidence of cognitive impairment [11], a greater cerebral small vessel disease score [34], an association with small vessel-related incidences of lacunar stroke [35], and a strong correlation to the fractional pressure loss across stenotic cerebral arteries [36]. A pulsatility index of > 1.1 has been used to attempt to predict cerebral microangiopathy in persons free of cerebral symptoms [37]. The interpretation of these data is that an increase in the pulsatility index reflects an increase in small vessel vascular resistance. This is similar to the present LDF pulsatility index interpretation since an increase in arteriolar resistance would be associated with a decrease in mean perfusion and an elevation in the pulsatile component. A variety of other types and uses of the pulsatility index have been investigated including for peripheral arterial disease [38,39], cardiac conditions [40-42], and obstetrics [43,44] to name only some. It is unclear if the LDF pulsatility index will provide as useful an index or benefit as these other uses. However, the fact that it is herein shown to be highly correlated with the PPG pulse amplitude opens the door to further exploratory studies in which this possibility may be investigated.

 Study limitations and recommendations

One study limitation is that the data was obtained from 10 subjects. Despite this, the number was sufficient to uncover significant correlations among the evaluated parameters and to provide a basis and baseline for further studies. Another aspect that may be viewed as a limitation is the demographics of the subjects evaluated. They were young healthy adults. Questions remain as to whether similar patterns and relationships would be present in healthy adults who were considerably more mature. Such a comparative study would seem to be warranted. Moreover, the utility of the basic relationships herein observed to clinical usage remains to be determined. However, the central finding, that the PPG pulse amplitude is significantly correlated with actual blood perfusion, paves the way for targeted future investigations in selective patient populations. 

## Conclusions

The findings of this pilot study demonstrate that when finger PPG pulse amplitudes are measured in individual subjects there is a moderate-to-strong correlation between the PPG pulse amplitude and skin blood perfusion. This fact impacts the confidence in using the widely available PPG parameter, peripheral perfusion index, as an indicator of changes in tissue perfusion. The findings also indicate that a related parameter, the LDF pulsatility index, is also highly correlated with the PPG pulse amplitude and may serve as a useful parameter for future clinical investigations. 
